# Supplementary material for: A Likelihood Approach for Real-Time Calibration of Stochastic Compartmental Epidemic Models
Source: PLoS Comput Biol. 2017 Jan 17;13(1):e1005257. doi: 10.1371/journal.pcbi.1005257 (PMC5240920; doi:10.1371/journal.pcbi.1005257)
Supplement: S1 File — (TAR.GZ) [file pcbi.1005257.s014.tar.gz › HSPH_Online-SI-Revision/EnKF07/n10-extreme/n10-extreme_EnKF2_table-4.pdf]

1	newly 3 weeks spec	1350.	139.38	{12., 187.}	91.843	1.32659	newly 3 weeks cumul	2770.	2049.51	{716., 5684.}	68.3765	0.39385	newly 5 weeks spec	1264.	35.09	{0., 34.}	97.6432	2.68825	newly 5 weeks cumul	5455.	2149.4	{729., 5688.}	69.6125	0.587249	newly 62 weeks cumul	8628.	2204.08	{729., 5688.}	77.1414	0.760867
2	newly 3 weeks spec	604.	111.65	{5., 374.}	84.3129	1.20644	newly 3 weeks cumul	1548.	603.34	{30., 1624.}	80.8902	0.84157	newly 5 weeks spec	678.	47.92	{0., 102.}	92.9322	1.81686	newly 5 weeks cumul	2970.	723.4	{35., 2128.}	81.0222	1.00946	newly 58 weeks cumul	6502.	835.67	{42., 2799.}	87.1475	1.27235
3	newly 3 weeks spec	1586.	631.9	{90., 1607.}	66.9117	0.612475	newly 3 weeks cumul	3187.	4855.44	{1170., 11 223.}	97.7446	0.344305	newly 5 weeks spec	1325.	157.4	{6., 359.}	88.1208	1.29956	newly 5 weeks cumul	6258.	5313.02	{1230., 11 427.}	61.97	0.378235	newly 64 weeks cumul	8818.	5556.91	{1313., 11 787.}	52.3115	0.398651
4	newly 3 weeks spec	888.	93.25	{10., 102.}	90.2736	1.33285	newly 3 weeks cumul	2367.	538.46	{52., 530.}	85.4271	0.992626	newly 5 weeks spec	862.	33.94	{2., 65.}	96.0626	1.84573	newly 5 weeks cumul	4124.	628.44	{71., 607.}	85.8007	1.13577	newly 58 weeks cumul	7204.	694.05	{91., 885.}	90.3658	1.31944
5	newly 3 weeks spec	1149.	87.01	{0., 89.}	92.7842	2.22257	newly 3 weeks cumul	2806.	873.31	{183., 1346.}	77.6041	0.741541	newly 5 weeks spec	1043.	25.49	{0., 26.}	97.5561	2.86855	newly 5 weeks cumul	5000.	945.3	{201., 1413.}	82.4904	0.945038	newly 61 weeks cumul	8544.	977.17	{202., 1424.}	88.5631	1.16201
6	newly 3 weeks spec	1137.	182.56	{11., 375.}	87.1082	1.20909	newly 3 weeks cumul	2504.	2297.88	{531., 6254.}	75.7396	0.383623	newly 5 weeks spec	1175.	46.38	{0., 65.}	96.5396	2.57525	newly 5 weeks cumul	4971.	2429.41	{531., 6264.}	67.2511	0.540893	newly 62 weeks cumul	8740.	2502.82	{531., 6264.}	74.7281	0.739116
7	newly 3 weeks spec	962.	269.46	{0., 759.}	77.8981	1.64726	newly 3 weeks cumul	2111.	3709.29	{736., 8391.}	115.365	0.392633	newly 5 weeks spec	1027.	79.99	{0., 264.}	92.9416	2.42388	newly 5 weeks cumul	4213.	3923.02	{749., 9838.}	64.1348	0.393896	newly 63 weeks cumul	7880.	4111.89	{777., 10 438.}	59.0138	0.513364
8	newly 3 weeks spec	963.	290.11	{2., 642.}	99.9782	1.6957	newly 3 weeks cumul	2334.	956.21	{27., 2566.}	95.0056	1.13059	newly 5 weeks spec	978.	88.32	{0., 465.}	90.9693	2.57749	newly 5 weeks cumul	4380.	1223.55	{29., 3894.}	91.1048	1.32113	newly 60 weeks cumul	8557.	1328.52	{31., 4476.}	85.8754	1.53764
9	newly 3 weeks spec	987.	272.26	{4., 638.}	97.5785	1.58775	newly 3 weeks cumul	2373.	854.48	{122., 2234.}	89.716	0.978976	newly 5 weeks spec	932.	107.19	{0., 618.}	88.5612	2.43648	newly 5 weeks cumul	4333.	1158.2	{127., 2750.}	89.0916	1.16451	newly 60 weeks cumul	8041.	1308.07	{129., 4541.}	85.3781	1.36588
10	newly 3 weeks spec	784.	254.51	{3., 507.}	101.445	1.58532	newly 3 weeks cumul	1916.	929.51	{57., 2240.}	101.411	0.986373	newly 5 weeks spec	979.	87.32	{0., 261.}	91.2748	2.50272	newly 5 weeks cumul	3879.	1181.17	{59., 2376.}	94.1307	1.20231	newly 60 weeks cumul	8180.	1305.51	{59., 4018.}	87.0921	1.44462
11	newly 3 weeks spec	908.	292.87	{6., 684.}	92.9725	1.34192	newly 3 weeks cumul	2169.	1354.89	{34., 4106.}	105.19	1.01932	newly 5 weeks spec	841.	112.71	{2., 146.}	90.2842	1.61669	newly 5 weeks cumul	3950.	1648.6	{43., 5938.}	94.6228	1.13904	newly 55 weeks cumul	7949.	1836.16	{58., 6659.}	87.5524	1.31526
12	newly 3 weeks spec	811.	244.4	{2., 384.}	101.998	1.66395	newly 3 weeks cumul	1803.	705.73	{12., 2116.}	93.5502	1.08787	newly 5 weeks spec	1021.	95.48	{0., 282.}	90.7365	2.39951	newly 5 weeks cumul	3891.	974.59	{16., 2243.}	91.5921	1.32702	newly 60 weeks cumul	7914.	1098.42	{16., 2623.}	87.5388	1.56401
13	newly 3 weeks spec	841.	178.43	{5., 366.}	88.8977	1.26242	newly 3 weeks cumul	2317.	1001.75	{172., 1606.}	91.271	0.815226	newly 5 weeks spec	848.	82.61	{0., 119.}	94.487	1.93306	newly 5 weeks cumul	4080.	1206.84	{175., 1923.}	89.5186	0.975973	newly 56 weeks cumul	7098.	1360.97	{175., 2071.}	88.2087	1.14998
14	newly 3 weeks spec	971.	85.05	{0., 89.}	92.4768	2.26982	newly 3 weeks cumul	2497.	852.5	{174., 1402.}	77.1013	0.716758	newly 5 weeks spec	946.	23.08	{0., 18.}	97.5603	2.95897	newly 5 weeks cumul	4507.	920.16	{196., 1402.}	81.8997	0.924695	newly 61 weeks cumul	7771.	949.93	{196., 1402.}	87.776	1.14403
15	newly 3 weeks spec	832.	98.	{8., 230.}	89.2788	1.31997	newly 3 weeks cumul	2173.	537.65	{39., 758.}	84.4017	0.993341	newly 5 weeks spec	838.	39.76	{1., 78.}	95.2554	1.89512	newly 5 weeks cumul	3908.	638.31	{48., 1274.}	85.2607	1.14822	newly 58 weeks cumul	6416.	751.39	{53., 1788.}	88.2888	1.29328
16	newly 3 weeks spec	1062.	52.04	{0., 68.}	95.5989	2.32723	newly 3 weeks cumul	2353.	843.2	{180., 1515.}	77.8725	0.714018	newly 5 weeks spec	1082.	14.18	{0., 13.}	98.6895	3.04043	newly 5 weeks cumul	4527.	884.98	{187., 1804.}	83.5096	0.953046	newly 61 weeks cumul	8363.	902.8	{189., 1979.}	89.3615	1.20396
17	newly 3 weeks spec	1033.	314.92	{0., 774.}	77.5411	1.58084	newly 3 weeks cumul	2323.	3548.82	{746., 8881.}	92.5062	0.334398	newly 5 weeks spec	1116.	124.34	{0., 332.}	91.1487	2.25398	newly 5 weeks cumul	4627.	3867.72	{765., 10 091.}	62.5438	0.392434	newly 63 weeks cumul	8302.	4124.03	{809., 10 820.}	61.0372	0.51419
18	newly 3 weeks spec	1151.	64.69	{0., 76.}	94.4822	2.44819	newly 3 weeks cumul	2756.	833.74	{174., 1393.}	79.4681	0.767595	newly 5 weeks spec	1119.	20.93	{0., 17.}	98.1296	3.13692	newly 5 weeks cumul	5058.	891.37	{189., 1411.}	85.1208	0.994188	newly 61 weeks cumul	8663.	922.93	{195., 1411.}	89.6226	1.2111
19	newly 3 weeks spec	1098.	228.14	{0., 742.}	81.6357	1.82091	newly 3 weeks cumul	2182.	3497.6	{729., 7629.}	95.6187	0.311803	newly 5 weeks spec	1350.	68.59	{0., 276.}	94.9193	2.65738	newly 5 weeks cumul	4959.	3684.36	{757., 7629.}	58.2964	0.37785	newly 63 weeks cumul	8373.	3816.52	{761., 7629.}	62.0662	0.519354
20	newly 3 weeks spec	902.	262.35	{3., 490.}	99.4113	1.63833	newly 3 weeks cumul	2235.	952.24	{42., 2270.}	97.9973	1.0471	newly 5 weeks spec	986.	84.1	{0., 268.}	91.4706	2.52915	newly 5 weeks cumul	4273.	1199.79	{46., 2342.}	92.7594	1.24562	newly 60 weeks cumul	8476.	1308.05	{50., 4044.}	87.3833	1.4704
21	newly 3 weeks spec	966.	52.43	{0., 92.}	94.5725	2.12998	newly 3 weeks cumul	2273.	1238.02	{114., 2545.}	106.011	0.960535	newly 5 weeks spec	836.	30.51	{0., 70.}	96.6758	2.69701	newly 5 weeks cumul	4121.	1305.17	{115., 4034.}	92.5817	1.09615	newly 57 weeks cumul	7668.	1487.56	{115., 4034.}	87.091	1.22142
22	newly 3 weeks spec	1428.	567.23	{104., 999.}	67.5819	0.609814	newly 3 weeks cumul	2568.	3939.38	{1169., 9361.}	93.1285	0.320373	newly 5 weeks spec	1622.	175.49	{6., 384.}	89.1806	1.35176	newly 5 weeks cumul	6049.	4412.54	{1333., 10 768.}	60.2949	0.401388	newly 64 weeks cumul	9138.	4752.48	{1342., 11 200.}	56.9634	0.466411
23	newly 3 weeks spec	671.	217.34	{0., 728.}	89.699	1.6471	newly 3 weeks cumul	1760.	1532.53	{20., 7731.}	127.418	1.00798	newly 5 weeks spec	667.	57.73	{0., 42.}	95.3598	2.44872	newly 5 weeks cumul	3172.	1689.36	{22., 7916.}	102.174	1.14306	newly 59 weeks cumul	5972.	1804.78	{22., 7927.}	85.8778	1.3072
24	newly 3 weeks spec	922.	47.25	{0., 98.}	94.9403	2.21077	newly 3 weeks cumul	2515.	1096.32	{49., 3013.}	99.1412	1.0456	newly 5 weeks spec	778.	27.34	{0., 60.}	97.1388	2.7927	newly 5 weeks cumul	4256.	1154.47	{62., 3340.}	90.3654	1.16492	newly 57 weeks cumul	7579.	1294.31	{103., 3340.}	87.5953	1.28944
25	newly 3 weeks spec	1376.	115.6	{2., 156.}	95.5247	2.01742	newly 3 weeks cumul	2783.	543.84	{13., 628.}	93.0471	1.31222	newly 5 weeks spec	1327.	42.06	{0., 44.}	96.8304	2.81782	newly 5 weeks cumul	5644.	658.83	{14., 944.}	93.5239	1.56141	newly 60 weeks cumul	8660.	733.42	{14., 1867.}	93.3538	1.71452
26	newly 3 weeks spec	1193.	321.96	{0., 755.}	78.0771	1.59756	newly 3 weeks cumul	2728.	3544.62	{735., 8897.}	78.9076	0.338797	newly 5 weeks spec	913.	126.66	{0., 329.}	90.1446	2.15567	newly 5 weeks cumul	4845.	3868.89	{756., 10 107.}	62.1777	0.408775	newly 63 weeks cumul	6836.	4127.78	{780., 10 864.}	59.3616	0.460062
27	newly 3 weeks spec	1034.	55.8	{0., 70.}	95.2128	2.30873	newly 3 weeks cumul	2307.	762.79	{178., 1388.}	75.378	0.702377	newly 5 weeks spec	1086.	16.61	{0., 16.}	98.4705	3.1029	newly 5 weeks cumul	4510.	809.69	{178., 1403.}	83.194	0.952891	newly 61 weeks cumul	8570.	830.55	{184., 1403.}	90.3086	1.22108
28	newly 3 weeks spec	1424.	646.74	{67., 2034.}	68.2051	0.620992	newly 3 weeks cumul	2886.	4727.72	{1020., 10 325.}	107.974	0.360538	newly 5 weeks spec	1362.	181.43	{4., 372.}	86.6791	1.35106	newly 5 weeks cumul	5761.	5236.67	{1065., 11 654.}	65.8655	0.386507	newly 64 weeks cumul	8981.	5544.86	{1065., 11 833.}	53.3669	0.424636
29	newly 3 weeks spec	1269.	160.53	{0., 603.}	91.8668	1.8548	newly 3 weeks cumul	2814.	1275.56	{29., 5667.}	101.849	1.11564	newly 5 weeks spec	1154.	33.24	{0., 36.}	97.1196	2.79487	newly 5 weeks cumul	5251.	1372.92	{31., 5784.}	89.6812	1.28471	newly 59 weeks cumul	8475.	1469.15	{31., 6573.}	86.3352	1.42928
30	newly 3 weeks spec	975.	192.87	{1., 734.}	88.3497	1.64751	newly 3 weeks cumul	2275.	1384.21	{22., 6728.}	107.292	1.06171	newly 5 weeks spec	882.	47.06	{0., 39.}	96.1406	2.61843	newly 5 weeks cumul	4197.	1517.09	{24., 6938.}	91.3793	1.21532	newly 59 weeks cumul	7393.	1627.91	{24., 7855.}	83.155	1.37309
31	newly 3 weeks spec	1130.	171.14	{12., 356.}	88.1062	1.18427	newly 3 weeks cumul	2628.	2195.05	{817., 5674.}	71.1884	0.378038	newly 5 weeks spec	1066.	38.21	{0., 51.}	97.2448	2.37432	newly 5 weeks cumul	4862.	2308.78	{881., 5974.}	68.6697	0.537654	newly 62 weeks cumul	8496.	2360.07	{924., 6045.}	76.0806	0.738323
32	newly 3 weeks spec	826.	219.93	{23., 341.}	85.9818	0.962231	newly 3 weeks cumul	2183.	957.08	{133., 1222.}	80.6908	0.673711	newly 5 weeks spec	784.	113.3	{3., 220.}	88.2551	1.41823	newly 5 weeks cumul	3849.	1226.44	{175., 1667.}	82.5861	0.805427	newly 54 weeks cumul	7242.	1562.	{204., 2810.}	82.7595	0.965699
33	newly 3 weeks spec	1646.	144.75	{10., 175.}	92.6154	1.43684	newly 3 weeks cumul	3764.	1926.71	{531., 4001.}	68.4843	0.496368	newly 5 weeks spec	1179.	37.83	{0., 29.}	97.2867	2.65185	newly 5 weeks cumul	6592.	2035.7	{532., 4077.}	73.4014	0.677174	newly 62 weeks cumul	8743.	2092.6	{541., 4260.}	78.617	0.78863
34	newly 3 weeks spec	1346.	555.27	{66., 1157.}	69.0899	0.67943	newly 3 weeks cumul	2950.	4469.93	{1129., 11 125.}	91.5088	0.321215	newly 5 weeks spec	1080.	135.18	{2., 358.}	87.7593	1.49555	newly 5 weeks cumul	5397.	4864.47	{1252., 11 507.}	63.9524	0.369758	newly 64 weeks cumul	7875.	5111.45	{1288., 11 514.}	56.5319	0.416094
35	newly 3 weeks spec	787.	194.37	{1., 726.}	88.2351	1.65427	newly 3 weeks cumul	2046.	1485.77	{44., 7668.}	113.129	0.978829	newly 5 weeks spec	782.	58.57	{0., 48.}	94.8197	2.54274	newly 5 weeks cumul	3686.	1640.	{44., 7864.}	94.5567	1.12129	newly 59 weeks cumul	7161.	1782.16	{44., 7897.}	82.6035	1.30034
36	newly 3 weeks spec	900.	77.72	{0., 93.}	93.0489	1.89125	newly 3 weeks cumul	2289.	1245.48	{118., 2725.}	105.561	0.940121	newly 5 weeks spec	946.	48.43	{0., 68.}	96.1533	2.62144	newly 5 weeks cumul	4201.	1347.62	{118., 4163.}	92.6736	1.0793	newly 57 weeks cumul	8382.	1640.47	{118., 5172.}	86.0681	1.2225
37	newly 3 weeks spec	1140.	275.55	{4., 661.}	95.4325	1.65361	newly 3 weeks cumul	2692.	877.26	{115., 2478.}	88.6768	1.04328	newly 5 weeks spec	1097.	102.18	{0., 584.}	90.6855	2.50721	newly 5 weeks cumul	4963.	1172.83	{122., 3909.}	87.9273	1.23502	newly 60 weeks cumul	8552.	1303.84	{122., 4563.}	85.9062	1.41614
38	newly 3 weeks spec	1819.	239.11	{0., 728.}	86.8549	1.82803	newly 3 weeks cumul	3439.	3397.81	{716., 7366.}	59.6031	0.305404	newly 5 weeks spec	1286.	76.23	{0., 300.}	94.0723	2.48156	newly 5 weeks cumul	6558.	3602.29	{736., 7366.}	56.9175	0.4349	newly 63 weeks cumul	8641.	3753.55	{740., 7366.}	61.2414	0.510973
39	newly 3 weeks spec	1549.	309.62	{0., 791.}	80.5771	1.67187	newly 3 weeks cumul	3391.	3767.63	{746., 10 134.}	76.4185	0.397734	newly 5 weeks spec	1272.	91.26	{0., 290.}	93.0031	2.3305	newly 5 weeks cumul	6224.	4013.7	{767., 10 547.}	63.3515	0.493475	newly 63 weeks cumul	8953.	4236.05	{767., 11 456.}	61.9054	0.553408
40	newly 3 weeks spec	954.	274.18	{5., 627.}	98.7799	1.56841	newly 3 weeks cumul	2231.	872.77	{118., 2260.}	91.866	0.986668	newly 5 weeks spec	887.	103.34	{0., 608.}	88.4825	2.41628	newly 5 weeks cumul	4133.	1168.27	{121., 3299.}	90.1551	1.17503	newly 60 weeks cumul	7710.	1330.23	{121., 5825.}	85.2313	1.37305
41	newly 3 weeks spec	1055.	214.96	{0., 691.}	90.8815	1.8466	newly 3 weeks cumul	2675.	1516.18	{33., 7667.}	105.344	1.12159	newly 5 weeks spec	964.	64.92	{0., 46.}	94.3734	2.70803	newly 5 weeks cumul	4723.	1691.16	{34., 7932.}	91.5109	1.26561	newly 59 weeks cumul	8244.	1838.36	{34., 7962.}	83.3013	1.41799
42	newly 3 weeks spec	812.	159.07	{24., 325.}	83.5776	0.930317	newly 3 weeks cumul	2241.	917.25	{271., 1579.}	77.1232	0.630407	newly 5 weeks spec	727.	69.47	{0., 157.}	91.0358	1.50548	newly 5 weeks cumul	3835.	1089.16	{294., 1972.}	80.2216	0.763008	newly 54 weeks cumul	7356.	1295.81	{310., 2375.}	83.8085	0.951768
43	newly 3 weeks spec	1345.	548.6	{90., 1017.}	65.8677	0.582932	newly 3 weeks cumul	2361.	4005.6	{1149., 9194.}	100.083	0.309248	newly 5 weeks spec	1603.	159.23	{5., 367.}	90.0667	1.40289	newly 5 weeks cumul	5687.	4444.31	{1255., 10 449.}	55.3921	0.34587	newly 64 weeks cumul	8914.	4705.78	{1255., 10 676.}	54.5497	0.426908
44	newly 3 weeks spec	783.	192.99	{0., 229.}	98.4278	1.89565	newly 3 weeks cumul	2116.	1329.14	{38., 4664.}	102.924	0.969855	newly 5 weeks spec	810.	77.74	{0., 97.}	93.4741	2.19287	newly 5 weeks cumul	3802.	1530.45	{47., 6349.}	92.9193	1.10112	newly 55 weeks cumul	7747.	1663.94	{62., 6811.}	86.6186	1.29265
45	newly 3 weeks spec	892.	198.68	{1., 665.}	91.1076	1.72473	newly 3 weeks cumul	2335.	1372.3	{32., 7596.}	104.998	1.02357	newly 5 weeks spec	873.	51.13	{0., 44.}	95.6483	2.62321	newly 5 weeks cumul	4172.	1515.98	{35., 7765.}	91.7804	1.17605	newly 59 weeks cumul	7795.	1622.1	{38., 7771.}	83.4119	1.35532
46	newly 3 weeks spec	811.	73.14	{0., 93.}	93.1566	1.89435	newly 3 weeks cumul	2110.	1223.03	{115., 2577.}	108.135	0.920877	newly 5 weeks spec	861.	49.2	{0., 72.}	95.7747	2.57031	newly 5 weeks cumul	3857.	1327.19	{115., 4000.}	93.8711	1.05506	newly 57 weeks cumul	7935.	1625.96	{115., 4884.}	86.4877	1.20952
47	newly 3 weeks spec	1061.	177.95	{1., 689.}	89.5278	1.71258	newly 3 weeks cumul	2374.	1537.73	{25., 7700.}	113.149	1.07185	newly 5 weeks spec	1104.	33.13	{0., 40.}	96.9991	2.664	newly 5 weeks cumul	4682.	1638.71	{34., 7924.}	91.6999	1.23805	newly 59 weeks cumul	8780.	1704.8	{35., 7935.}	83.9642	1.42083
48	newly 3 weeks spec	1085.	134.72	{11., 169.}	90.8406	1.2322	newly 3 weeks cumul	2381.	2061.95	{653., 6324.}	71.6342	0.362012	newly 5 weeks spec	1180.	31.66	{0., 29.}	97.9593	2.67522	newly 5 weeks cumul	4857.	2154.34	{741., 6334.}	69.2703	0.553384	newly 62 weeks cumul	8650.	2204.19	{759., 6334.}	77.2064	0.764759
49	newly 3 weeks spec	1202.	264.55	{0., 751.}	80.515	1.69983	newly 3 weeks cumul	2426.	3403.77	{717., 7623.}	81.0375	0.302891	newly 5 weeks spec	1386.	84.27	{0., 299.}	93.9199	2.55797	newly 5 weeks cumul	5269.	3629.85	{734., 7623.}	58.2397	0.400861	newly 63 weeks cumul	8545.	3782.85	{741., 7688.}	62.8743	0.538048
50	newly 3 weeks spec	1062.	55.9	{0., 67.}	95.4275	2.42107	newly 3 weeks cumul	2104.	850.59	{178., 1710.}	71.6573	0.621035	newly 5 weeks spec	1231.	16.62	{0., 10.}	98.6499	3.24447	newly 5 weeks cumul	4630.	897.65	{188., 2000.}	81.7968	0.914946	newly 61 weeks cumul	8721.	920.58	{188., 2000.}	89.4441	1.17952
